# Expression of *ZmGA20ox* cDNA alters plant morphology and increases biomass production of switchgrass (*Panicum virgatum* L.)

**DOI:** 10.1111/pbi.12514

**Published:** 2016-01-23

**Authors:** Phat T. Do, Joann R. De Tar, Hyeyoung Lee, Michelle K. Folta, Zhanyuan J. Zhang

**Affiliations:** ^1^ Plant Transformation Core FacilityDivision of Plant SciencesUniversity of MissouriColumbiaMOUSA

**Keywords:** *gibberellin*, *gibberellin 20‐oxidase*, biofuel, biomass, switchgrass

## Abstract

Switchgrass (*Panicum virgatum* L.) is considered a model herbaceous energy crop for the USA, for its adaptation to marginal land, low rainfall and nutrient‐deficient soils; however, its low biomass yield is one of several constraints, and this might be rectified by modulating plant growth regulator levels. In this study, we have determined whether the expression of the *Zea mays gibberellin 20‐oxidase* (*ZmGA20ox*) cDNA in switchgrass will improve biomass production. The *ZmGA20ox* gene was placed under the control of constitutive CaMV35S promoter with a strong TMV omega enhancer, and introduced into switchgrass via *Agrobacterium*‐mediated transformation. The transgene integration and expression levels of *ZmGA20ox* in T0 plants were analysed using Southern blot and qRT‐PCR. Under glasshouse conditions, selected transgenic plants exhibited longer leaves, internodes and tillers, which resulted in twofold increased biomass. These phenotypic alterations correlated with the levels of transgene expression and the particular gibberellin content. Expression of *ZmGA20ox* also affected the expression of genes coding for key enzymes in lignin biosynthesis. Our results suggest that the employment of ectopic *ZmGA20ox* and selection for natural variants with high level expression of endogenous *GA20ox* are appropriate approaches to increase biomass production of switchgrass and other monocot biofuel crops.

## Introduction

Biofuels are an important component of the energy sources of the planet, and there is great need for developing biofuel feedstock crops (Carroll and Somerville, 2009; Sticklen, 2008). Switchgrass (*Panicum virgatum* L.) was the first plant selected for bioenergy by U.S. Department of Energy in the 1990s (McLaughlin and Kszos, 2005). This perennial C4 grass has high productivity across different environments and is adapted to marginal land, low rainfall regions and nutrient‐deficient soil (Fike *et al*., 2006). Switchgrass produces net positive renewable energy and has positive environmental benefits (Schmer *et al*., 2008). U.S. Department of Energy and U.S. Department of Agriculture have projected a national goal for biofuel to supply 20% transportation fuels by 2030. About 1 billion dry tons of biomass will be annually required for the goal, of which one‐third of the biomass will come from perennial feedstock such as switchgrass. Therefore, increasing biofuel crop yields is a major goal of U.S. biomass energy research programme (Sanderson *et al*., 2006). Numerous factors affecting plant biomass production have been studied and applied in attempts to gain higher crop vegetative yields (Demura and Ye, 2010). Of these, manipulation of endogenous plant hormone contents is one of the most effective ways to improve plant growth, development and biomass.

Gibberellins (GAs) comprise a large family of diterpenoid carboxylic acids of more than one hundred compounds currently known in higher plants, fungi and bacteria (Hedden and Phillips, 2000; Olszewski *et al*., 2002; Yamaguchi *et al*., 2014, 2008). In higher plants, GA_1_, GA_3_, GA_4_ and GA_7_ are the most common active GAs that control diverse processes of plant growth and development. The complex pathways of bioactive GA biosynthesis in higher plant require three different classes of enzymes and the participation of different cell components (Olszewski *et al*., 2002; Yamaguchi *et al*., 2014). In the cytoplasm, the last steps of GA biosynthesis are catalysed by GA20‐oxidase (GA20ox) and GA3‐oxidase (GA3ox) to form various GA intermediates and mature bioactive GAs (Hedden and Phillips, 2000; Olszewski *et al*., 2002). GA20‐oxidase, a multifunctional enzyme, catalyses several sequential reactions in the formation of inactive gibberellins (GA_9_, GA_20_); then, GA3ox introduces a 3β‐hydroxyl group to form the mature products (Yamaguchi *et al*., 2014).

GA20ox is encoded by genes that have been cloned from various dicots (Carrera *et al*., 2000; Eriksson *et al*., 2000; Kang *et al*., 1999; Phillips *et al*., 1995) and monocots (Du *et al*., 2009; Toyomasu *et al*., 1997). The ectopic expression of genes coding for GA20ox has been shown to increase the levels of bioactive GAs and to affect plant growth and morphology. For example, overexpression of *GA20ox* caused a higher level of GA_4_ in *Arabidopsis thaliana* and consequently to accelerate elongated hypocotyls of seedlings, increasing shoot growth and early flowering (Croker *et al*., 1999). Overexpression of *GA20ox* in potato resulted in taller plants and longer leaf petioles (Carrera *et al*., 2000). Ectopic expression of *Arabidopsis GA20ox* increased bioactive GAs in transgenic tobacco, leading to increased plant growth and biomass production (Biemelt *et al*., 2004). In citrus, overexpression of *GA20ox* modified plant architecture. Transgenic citrus plants had much longer thorns and typical organs at juvenile stages. In addition, higher levels of active GA_1_ were also observed in these plants (Fagoaga *et al*., 2007). In hybrid aspen (Eriksson *et al*., 2000), ectopic expression of *GA20ox* gene increased growth rate and biomass, and caused more and longer fibres compared to wild‐type plants.

In rice, Ayano *et al*. (2014) showed that expression of *GA20ox* correlated with GA_1_ and GA_4_ content and has a role in internode elongation, but most studies in monocots have focused on down‐regulation of *GA20ox* gene to reduce plant height and increase reproductive yields (Sasaki *et al*., 2002). Overall, these results indicate that altering the expression of *GA20ox* changes GA levels, and consequently also plant growth, development and biomass production.

Here, we report that the expression of *ZmGA20ox* cDNA in switchgrass results in elevation of bioactive GA levels and altered plant architecture with longer internodes, leaves and increased fresh and dry biomass. Moreover, the expression of *ZmGA20ox* was found to affect expression of genes in lignin biosynthesis. Our results suggested that ectopic *ZmGA20ox* is a viable approach for increased biomass for biofuel production by switchgrass and possibly other energy monocots.

## Results

### Generation of transgenic switchgrass plants with *ZmGA20ox*

**Figure 1:**
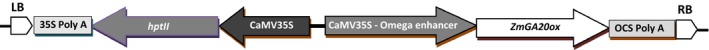
Schematic of the T‐DNA region of the binary construct for switchgrass transformation. 35S Poly A: CaMV35 poly A terminator; *hptII:* hygromycin phosphotransferase II gene; CaMV35S, CaMV35S promoter; *ZmGA20ox, Z. mays* Gibberellin (GA) 20‐oxidase; OCS poly A, octopine synthase terminator.

**Figure 2:**
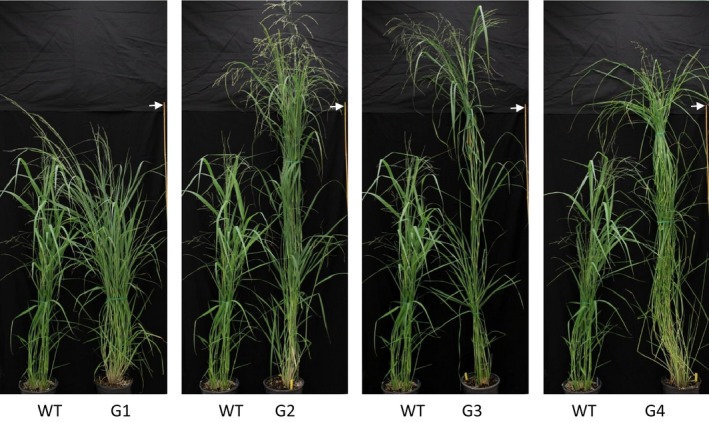
Switchgrass phenotypes. G1‐G4, transgenic groups 1–4, respectively; WT, wild‐type control. Arrow indicates height measurement (bar = 180 cm).

**Figure 3:**
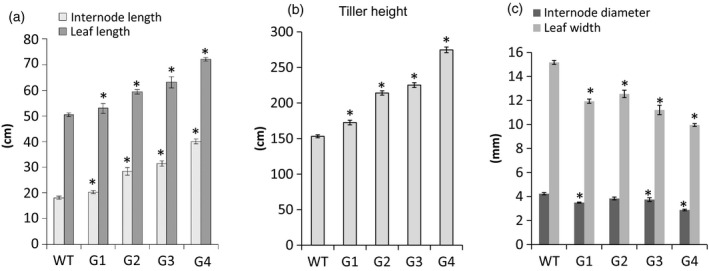
Morphology of T0 transgenic plants. G1‐G4, transgenic groups 1–4, respectively; WT, wild‐type control. *Significant difference at *P* < 0.05.

**Figure 4:**
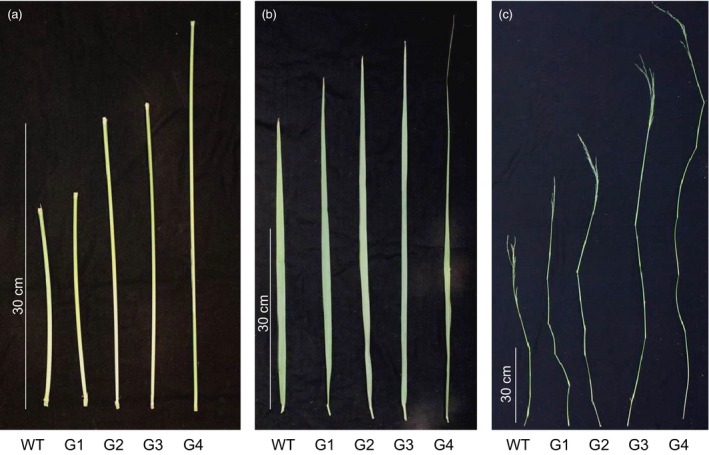
T0 plant morphology. (a) Internodes; (b) leaves; (c) tillers. G1‐G4, transgenic groups 1–4; WT, wild‐type.

Using binary construct for overexpression of *ZmGA20ox* and *Agrobacterium*‐mediated transformation, more than 20 transgenic switchgrass events were produced and confirmed based on the leaf painting and genomic PCR using specific primers for *ZmGA20ox* and *hptII* genes (Figures 1 and S1, and Table S1). After 2 months of growth under glasshouse conditions, phenotypic differences became obvious among the transgenic events compared to WT control plants. All transgenic events exhibited longer tillers (Figures 2, 3 and 4) and could be divided into four groups: group 1—more tillers but the same growth as WT; group 2—more tillers and much faster growth than WT; group 3—fewer tillers and much faster growth than WT; and group 4—very thin leaves and more tillers and much faster growth than WT. Groups 1–4 exhibited 12.7%, 39.8%, 47.1% and 80% increase in tiller height, respectively, as compared to WT (Figure 3). The longer tillers of transgenic plants were the results of longer internodes and leaves, typical of morphological changes caused by GA_3_. Group 1 plants displayed less change in internode and leaf elongation than remaining groups. Of all groups, group 4 had the largest increase in leaf (42.7%) and internode (approximately 120%) length (Figures 3 and 4).

All transgenic switchgrass plants exhibited a reduction in internode diameter and leaf width. The internode diameter of transgenic plants was reduced to between 2.90 mm and 3.84 mm compared to 4.23 mm of WT plants (Figures 3 and 4). The average leaf width of transgenic plants was between 10 mm (group 4) and 12.5 mm (group 2), whereas that of WT plants was 15.2 mm. There was a correlation between the increase in internode and leaf length and the reduction in internode diameter and leaf width of transgenic plants except for group 1. Transgenic plants of this group showed a significant reduction in internode diameter as compared to group 2. Interestingly, a substantial increase in the number of tillers appears to be compensated by the decreased internode diameter and leaf width. Other groups displayed similar growth phenotypes, that is the longer leaves and internodes were compensated by the narrow leaves and small internode diameters. In addition, group 4 exhibited a weak tiller phenotype that could not stand up well. Curling leaves also occurred in plants of this group.

### Effects of ectopic *ZmGA20ox* on growth rate, biomass and flowering time

*ZmGA20ox* transgenic switchgrass plants exhibited increased growth rate, especially during vegetative development stage, and faster tiller emergence and elongation were observed in all transgenic lines. For tiller number, groups 2, 3 and 4 transgenic and WT plants had no significant difference in the number of tillers of each plant. By contrast, group 1 transgenic plants had a remarkable increase in the number of tillers (by approximately 130%) compared to WT plants (Table 1). Interestingly, all transgenic groups showed increases in both fresh and dry biomass weight, but a 19.7%–34.8% reduction in fresh to dry weight ratios, respectively. Specifically, groups 1, 2 and 3 displayed a 1.8‐ to twofold increase in the whole dry biomass as compared to WT plants, whereas group 4 had insignificant increase in dry biomass. To reconfirm the faster growth of transgenic plants, the tillers were cut and the growth rates measured again. One month after cutting back, tillers of ectopic *ZmGA20ox* plants were 50.5%–86.9% higher than WT plants (Figure S2).

**Table 1:** Tiller number and biomass

Groups	Tiller number	Dry weight (g)	Fresh/dry ratio
WT	23.7	50.7	4.51
G1	54.5^a^	108.9^a^	3.01^a^
G2	31.5	103.1^a^	3.31^a^
G3	17.3	94.2^a^	2.94^a^
G4	28.8	80.6	3.62^a^

a Significance relative to wild‐type at *P* ≤ 0.05.

Switchgrass flowering was observed at the R1 stage of individual tiller (Hardin *et al*., 2013), but flowering times were not correlated with faster growth rate. At 12 weeks under glasshouse condition, wild‐type plants exhibited more than 17% flowering tillers, but for transgenic plants, that varied from 0% to 10.6% (Figure S4). Flowering tillers of wild‐type switchgrass reached a peak (96%–100%) at 17 weeks. However, the rate of flowering tillers of transgenic switchgrass gradually increased and ranged from 48.1% to 70.2%.

### Cell size changed in *ZmGA20ox* transgenic plants

**Figure 5:**
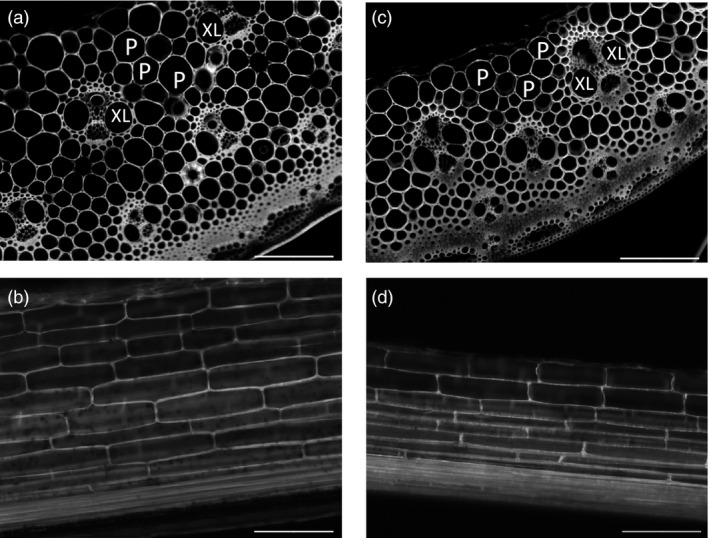
Fluorescent microscopy of plant tissues. P, pith cells; XL, xylem cells. (a) and (b). Cross and longitudinal sections of wild‐type internodes, respectively. (c) and (d). Cross and longitudinal sections of transgenic internodes, respectively. Bar: 200 μm at 10× magnification.

We examined the effect of *ZmGA20ox* overexpression on internode cell size of transgenic plants from group 4 using fluorescence microscopy (Figure 5). Both longitudinal and cross sections of transgenic plant (G4‐1) showed smaller pith and xylem cells, and transgenic xylem cells displayed 22.8% reduction in average cell size, while the reduction in pith cell size was 36.6% as compared to wild‐type plants (Table 2). These data were consistent with decreased tiller thickness and smaller internode diameter. There was no difference in the vascular bundle distribution in stems and the pith cell length between transgenic plants and wild‐type control (Figure 5 and Table 2). Both wild‐type and transgenic plants exhibited three circles of vascular bundles in the cross section of fully elongated internodes at the same developmental stage. Therefore, the longer internodes and leaves of *ZmGA20ox* transgenic plants could be caused by the increase in cell division at the position of leaf divisional zones and intercalary meristems.

**Table 2:** Cell measurements

Plants	Number of Pith cells/mm^2^	Pith cell length (μm)	Xylem cell size (μm^2^)
WT	440.31 ± 10.20	247.47 ± 6.3	3773.78 ± 73.24
G4‐1	694.62 ± 35.44^a^	243.43 ± 6.6	2914.67 ± 77.05^a^

a Significance relative to wild‐type at *P* ≤ 0.01.

### Transgenic phenotypes correspond to *GA20ox* transcript and GAs levels

**Figure 6:**
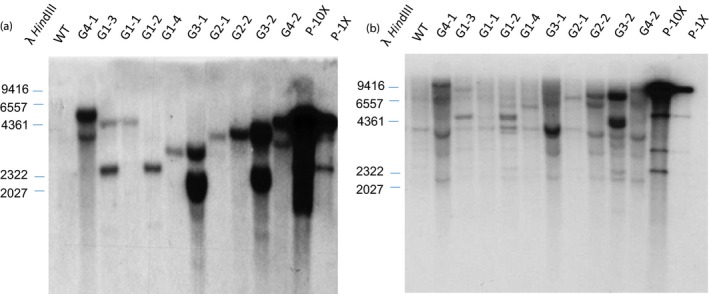
Southern blot analysis of T0 events using *hptII* probe (a) and *GA20ox* probe (b), WT, wild‐type control; G1‐G4, random samples from various groups; P‐10X and P‐1X, plasmid digestion representing 10× and 1× genome equivalents.

Transgene integration patterns of various phenotypic groups were analysed. Several events in each group were randomly selected for Southern blot using *ZmGA20ox* and *hptII* partial open‐reading frames as probes, respectively. Genomic DNA was digested with restriction enzyme *Bam*HI, which cuts once within the T‐DNA region, allowing identification of different events and estimation of the number of transgene copies (Figures 1 and 6). When the *hptII* probe was used, varying banding patterns were shown in transgenic samples, whereas no hybridizing band was detected in wild‐type plants (Figure 6a). However, when the *ZmGA20ox* probe was used, besides varying molecular weight bands corresponding to the transgene, a 4‐kb hybridizing band was shown in all samples including wild‐type plant. This band is believed to be the endogenous *GA20ox* gene which shares a high degree of sequence homology with *ZmGA20ox* (Figure 6b).

**Figure 7:**
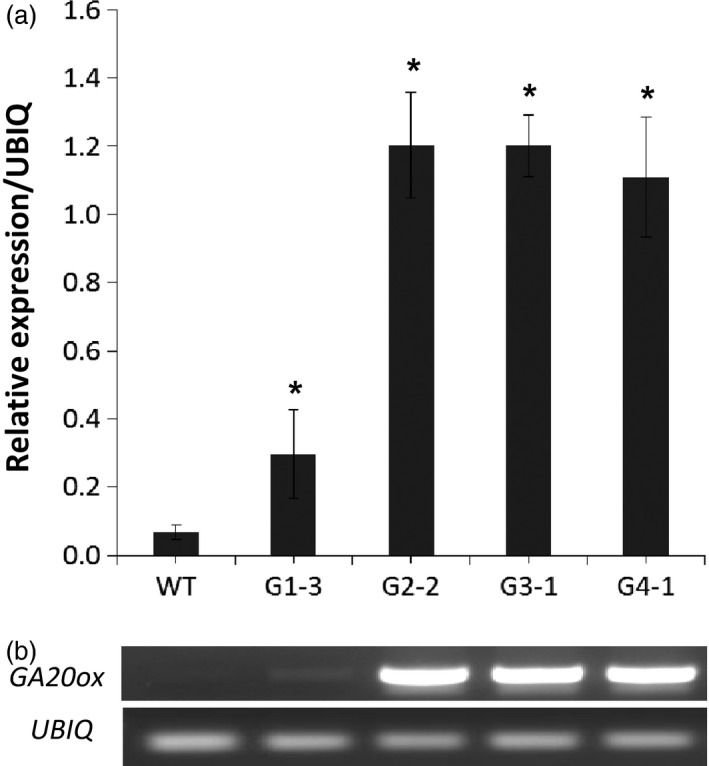
Transcript abundance of *GA20o*x in representative event of each group (G1‐3, G2‐2, G3‐1, and G4‐1) compared to wild‐type (WT). Quantitative real‐time PCR analysis of *GA20ox* transcript levels (a) and RT‐PCR gel analysis of *GA20ox* and *UBIQ* transcripts (b). *Significant relative to WT (*P* < 0.05).

We analysed transgene expression and GA levels using a representative event from each group. RNA samples isolated from whole tillers at elongation stage E1 of these events were used for cDNA synthesis, and then for both RT‐PCR and quantitative real‐time PCR (qRT‐PCR) with *GA20ox* primers (Table S1). The RT‐PCR and qRT‐PCR results were consistent, showing increases in the transcript abundance of transgenic events from the four main groups (Figure 7). Transgenic event G1‐3 showed 4.4‐fold increase in the transcription level of *GA20ox* compared to WT control, while that for other events ranged from 16.3‐ to 17.7‐fold. These results agreed with the phenotypic changes of these transgenic groups as described above. No significant difference in the transcript abundances was found between transgenic lines among groups 2, 3 and 4.

We then analysed GA in *ZmGA20ox* transgenic events, and focused on GA_1_ and GA_4_, two bioactive GAs in higher plants (Table 3). The levels of endogenous GAs were correlated to the degrees of altered transgenic phenotypes. Wild‐type and G1‐3 plants had very low levels of GA_1_ and GA_4_ that could not be detected by our current GAs detection system. A high concentration of GA_4_ (7.4 ng/g) was recorded, whereas GA_1_ was not detectable in transgenic event G2‐2. Both GA_1_ and GA_4_ were detected in transgenic events G3‐1 and G4‐1 at high levels. Importantly, the highest content of bioactive GAs occurred in G4‐1 transgenic plant that had the most significant alteration of phenotype. Furthermore, a higher concentration of GA_4_ than GA_1_ was found in all GA‐detectable transgenic plants.

**Table 3:** Concentration of bioactive gibberellins (GAs) in whole tiller at E1 stage^a^

GAs	WT	G1‐3	G2‐1	G3‐1	G4‐1
GA_1_	n.d.	n.d.	n.d.	1.2 ± 0.12	1.6 ± 0.12
GA_4_	n.d.	n.d.	7.4 ± 0.94	4.3 ± 0.40	10.5 ± 0.92

a Concentration in nanograms per gram fresh weight, as means of three independent measurements.

nd, not detectable.

### Effects of ectopic *ZmGA20ox* on lignin gene expression

**Figure 8:**
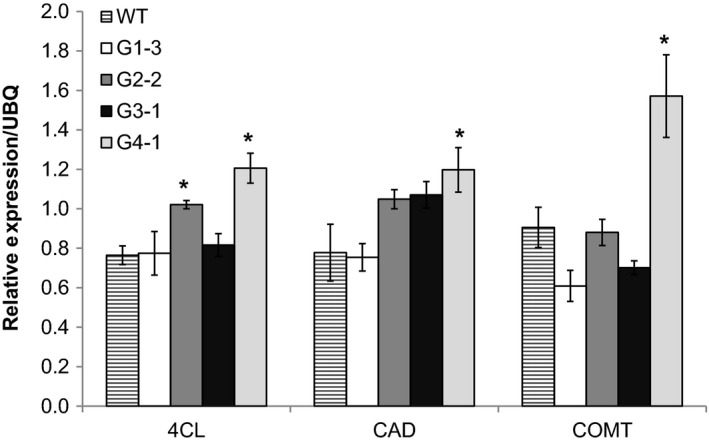
Relative expressions of lignin genes in transgenic switchgrass and wild‐type by qRT‐PCR. 4CL, 4‐coumarate:CoA ligase; CAD, cinnamyl alcohol dehygrogenase; COMT, caffeic acid 3‐*O*‐methyltransferase. *Significant relative to wild‐type (*P* < 0.05).

To explore whether changes in *GA20ox* expression affected lignin biosynthesis, transcripts of three genes coding for enzymes in lignin biosynthesis pathway were analysed by qRT‐PCR. Of these, 4CL (4‐coumarate: CoA ligase) is an enzyme in the early steps of this pathway, while cinnamyl alcohol dehygrogenase (CAD) and caffeic acid 3‐*O*‐methyltransferase (COMT) catalyse the final steps of monolignol biosynthesis. In group 4 transgenic plants (G4‐1), *GA20ox* expression level correlated with transcript levels of lignin genes, showing a significant increase in the transcript abundance of all three lignin genes (Figure 8). However, remaining groups had only a minor change in the expression levels of these lignin genes. For example, in group 2 plants (G2‐2), the expression of *4CL* gene was clearly increased, but *CAD* and *COMT* expressions had no significant change. The phloroglucinol‐HCl staining for lignin was associated with an altered expression of pathway‐specific genes (Figure S3). Much higher lignin accumulation was observed in internode cross sections of transgenic group 4, where lignin staining was exhibited not only in sclerenchyma cell walls but also clearly in parenchyma cell walls. Compared to wild‐type, transgenic group 1 did not show a clear change in lignin accumulation. In addition, groups 2 and 3 had a slight increase in lignin content.

## Discussion

GA20 oxidase is a key enzyme in the pathways of bioactive GA biosynthesis in higher plants, and its overexpression has been shown to alter plant phenotypes and to increase relative growth rates in many plant species. Biomass improvement by modulation of *GA20ox* genes has been achieved in some dicot plants such as tobacco and hybrid aspen (Biemelt *et al*., 2004; Eriksson *et al*., 2000). In this study, the observed alteration of plant architecture of transgenic switchgrass with the *ZmGA20ox* gene under the control of the CaMV35S promoter resembled that in dicots (Biemelt *et al*., 2004; Carrera *et al*., 2000; Croker *et al*., 1999; García‐Hurtado *et al*., 2012), maize (Voorend *et al*., 2015) as well as that of exogenous applications of GA_3_ to other monocots (Tsai and Arteca, 1985). These architecture changes included longer leaves, internodes and tillers but smaller leaves and internode diameters compared to wild‐type control plants. These morphological changes were highly corresponded with the expression of *GA20ox* and bioactive GA levels, which were higher than the wild‐type control. Furthermore, varied phenotypes of these groups were correlated to the different contents and forms of bioactive GAs, respectively. Finally, higher biomass and reduced dry‐fresh weight ratios were obtained in all transgenic plants. The present work is the first study to report the ectopic expression of *GA20ox* in switchgrass for improving biomass.

Bioactive GA_1_ and GA_4_ are produced at the final stage of GA biosynthesis catalysed through two parallel pathways, involving 13‐hydroxylation and the non‐13‐hydroxylation (Vidal *et al*., 2001). Higher contents of GA_1_ than GA_4_ were shown in the *GA20ox* overexpressing transgenic citrus (*Citrus sinensis*) (Fagoaga *et al*., 2007) and Populus (Eriksson *et al*., 2000). In contrast, ectopic *GA20ox* in potato (*Solanum tuberosum*) displayed much higher contents of GA_4_ in apical shoots (Carrera *et al*., 2000). The same result was obtained in both shoots and fruits of transgenic tomato (*Solanum lycopersicum*). The phenotypes of *GA20ox* transgenic tomato plants were due to the increase of bioactive GA_4_ content (García‐Hurtado *et al*., 2012), whereas both GA_4_ and GA_1_ had function in internode elongation of deepwater rice (Ayano *et al*., 2014). In the current study, the same altered phenotypes were exhibited by G2‐2 and G3‐1 lines, even though the lower content of GA_4_ but higher level of GA_1_ occurred in the G3‐1 line. In addition, the largest phenotypic alteration of transgenic line G4‐1 was consistent with the highest levels of GA_1_ and GA_4_ (Table 3). These data indicate that the elongated phenotypes of *ZmGA20ox* transgenic switchgrass resulted from the activation of both bioactive GAs.

Longer internodes in *CcGA20ox1* overexpression citrus could be correlated to cell divisions (Fagoaga *et al*., 2007). Similar results were observed in the transgenic *AtGA20ox* Populus trees (Eriksson *et al*., 2000). By contrast, transgenic *AtGA20ox* tobacco plants showed longer shoots that resulted from both cell divisions and elongation (Biemelt *et al*., 2004). The occurrence of cell division and elongation events corresponded to the regions of active GA biosynthesis and signalling (Kaneko *et al*., 2003). Moreover, in the study of GA biosynthesis and signal transduction in rice, Ayano *et al*. (2014) found that internode elongation was induced by the accumulation of GA during submergence. The activation of intercalary meristem located in the nodes was proposed as a driving force in internode elongation. GA levels were suggested to regulate the growth of maize leaves by spatial control of cell division (Nelissen *et al*., 2012). In our study, no difference in the length of pith cells between transgenic lines and wild‐type control plant was found. Therefore, the elongated internodes and leaves of *ZmGA20ox* transgenic plants were possibly consequences of the increased cell divisions in the leaf divisional zones, leaf primordia and intercalary meristems under higher levels of GAs (Figure 5 and Table 2).

Gibberellin deficiency by ectopic expression of *GA2ox* promoted earlier tiller formations, and higher tiller number was indicated in rice (Lo *et al*., 2008) and switchgrass (Wuddineh *et al*., 2015). However, the number of switchgrass tillers was not correlated with the GA levels. The semi‐dwarf switchgrass lines showed an increase in tiller number, whereas dwarf lines displayed a reduction in number of tillers per plant relative to wild‐type controls (Wuddineh *et al*., 2015). On the other hand, GAs were shown to increase tiller numbers of Welsh onion by initiating and promoting axillary bud development (Yamazaki *et al*., 2015). In addition, Ni *et al*. (2015) reported that GAs induced the formation of secondary buds as well as promoted shoot branching of some perennial woody plants by synergistically acts with cytokinin. In our study, there was no significant difference in tiller number between transgenic groups showing high levels of GAs and nontransgenic wild‐type plants. However, a remarkable increase in the number of tillers was observed in transgenic group 1, which showed a slight increase in *GA20ox* transcript abundance. To reconcile the effects upon switchgrass tillering by different levels of GA deficiency (semi‐dwarf and dwarf switchgrass) (Wuddineh *et al*., 2015), it is possible that switchgrass tiller formation and development may be impacted by different levels and components of bioactive GAs.

The overexpression of *GA20ox* in *Arabidopsis* promoted flowering (Blázquez and Weigel, 2000; Rieu *et al*., 2008). By contrast, GAs were indicated to inhibit flowering in grapevine (Boss and Thomas, 2002) and a similar observation was made in tomato (García‐Hurtado *et al*., 2012). Recently, Yamaguchi *et al*. (2014) reported that GA inhibits switch flower formation in *Arabidopsis* by interactions with genes promoting floral fate such as the EUI‐LIKE P450 A1 gene (ELA1), LEAFY transcription factor (LFY) and also DELLA proteins. The up‐regulation of ELA1 and LFY reduces the levels of GAs such as GA_4_. In our study, *ZmGA20ox* transgenic switchgrass exhibited a slight delay of flowering time compared to wild‐type control plants (Figure S4). Therefore, high levels of GAs, especially GA_4_, may negatively affect switchgrass flower formation. This speculation could be supported by the correlation between the slow flowering and the high GA_4_ contents in transgenic lines G2‐2 and G4‐1 (Figure S4 and Table 3). Moreover, no effect on flowering was observed in some plant species as a result of either GA abundance (Gallego‐Giraldo *et al*., 2007) or GA deficiency (Dijkstra *et al*., 2008). Therefore, the role of GAs on flowering varies and depends on the species. More research needs to be conducted to understand this complex mechanism.

Biomass yield and quality traits are two important criteria in selecting switchgrass for biofuel production. In our study, we found the association between the altered lignin gene expression, the lignin staining results and the levels of bioactive GAs in ectopic *ZmGA20ox* transgenic switchgrass. In transgenic group 4 (line G4‐1), the stronger lignin gene expression and histological staining correlated with the higher contents of GA_1_ and GA_4_. This observation is consistent with the results of lignin deposition study in tobacco under the *GA20ox* overexpression and different GA_3_ concentration treatments (Biemelt *et al*., 2004). Interestingly, in our study, some transgenic groups showed increased biomass production while displaying no significant change in lignin genes expression.

In summary, this is the first study on the effects of ectopic *GA20ox* expression on morphology and biomass of switchgrass. *ZmGA20ox* transgenic plants exhibited drastic alterations in plant phenotypes resulting in longer leaves and internodes. The increased growth rate caused increased fresh and dry biomass, and demonstrates a means to improve the biomass production of this feedstock and possible other cellulosic crops. Furthermore, the insignificant increase of lignin gene expression and lignin contents in those good phenotype groups should be desirable as bioenergy feedstock. Thus, the expression of ectopic *GA20 oxidase* could be a good experimental approach to benefit biomass production of monocot plants. Results from this study also implies that the use of variation in the natural *GA20ox* gene expression would be a viable means to select for improved varieties with higher biomass, avoiding the outcrossing risk of transgenic switchgrass pollen.

## Experimental procedures

### Vector construction and plant transformation

The open‐reading frame (1116 bp) of *Zea mays GA20 oxidase* (*ZmGA20ox*) from Genbank (NM_001112453.1) coding for 311 amino acids was synthesized by GenScript (Piscataway, NJ, USA). The *EcoR*I*/Hind*III fragment encompassing the *ZmGA20ox* gene and 35S promoter plus TMV Omega enhancer sequence was inserted into binary vector pCAMBIA1300 to generate transgenic T‐DNA construct (Figure 1), which was mobilized into *Agrobacterium tumefaciens* strain AGL1 for switchgrass transformation by the protocol of Li and Qu (2011) with modifications. Embryogenic calli were induced from mature seeds of switchgrass cultivar Alamo. Hygromycin B (Invitrogen^™^ Life Technologies, Carlsbad, CA, USA) was added to selected medium at 50 mg/L.

### Growth condition, leaf painting, sample collection and measurement

Transgenic switchgrass events were grown in glasshouses with day/night temperatures of 28/21°C, a photoperiod of 16 h light/8 h dark, in 3‐gal pots containing Promix soil supplemented by Osmocote (14‐14‐14) (Hummert International, Earth City, MO). Leaf painting was carried out by swiping 1 g/L hygromycin B solution onto the upper suffice of a leaf, and results were recorded 1 week later (Figure S1). The phenotypic data including the morphology of leaves, internodes and tillers were collected at R1 stage (Hardin *et al*., 2013). Internodes (I3 and I4) and their leaves were subjected to phenotypic observations. The above‐ground tissues were harvested when 50% of tillers reached R2 stage for biomass measurements. Dried weight was calculated after switchgrass samples were dried in an oven at 45°C for 96 h.

### PCR and Southern blot

Gene‐specific primers (Table S1) were used for PCRs to confirm the presence of transgenes, and Southern blots were used to confirm their integration into the genome. Genomic DNA was extracted from switchgrass leaf tissue using a CTAB procedure modified from (Dellaporta *et al*., 1983). For Southern blot analysis, 30 μg purified DNA was digested by a restriction enzyme that cut once within the T‐DNA region. Digested DNA fragments were fractionated on a 2.0% agarose gel prior to transfer to Zeta‐Probe^®^ GT nylon membrane (Bio‐Rad, Hercules, CA, USA). DNA was fixed to nylon membrane by UV cross‐link. Hybridization and membrane washing were conducted based on the Zeta‐Probe^®^ GT manufacturer's instructions at 65°C. Prime‐It^®^ RmT Random Primer Labeling Kit (Stratagene, La Jolla, CA, USA) was used to generate 32P‐labelled probes of *ZmGA20ox* (from synthetic transgene template) or *hptII* (from pCAMBIA1300 vector).

### Quantitative real‐time PCR

Total RNA was extracted from a whole tiller of wild‐type and transgenic switchgrass plants at elongation E1 stage (Hardin *et al*., 2013) using TRIZOL^®^ Reagent according to the manufacture's protocol (Invitrogen^™^ Life Technologies). The isolated RNA was treated with DNase‐I (Invitrogen^TM^ Life Technologies) to remove genomic DNA contamination. The first‐strand cDNA was synthesized from the DNase‐treated RNA using M‐MLV Reverse Transcriptase and Oligo‐dT primer (Promega, Madison, WI, USA). RT‐PCR was carried out using specific primers for *ZmGA20ox* gene (Table S1). qRT‐PCR was conducted using iQ^™^ SYBR^®^ Green Supermix (Bio‐Rad). The data were normalized using the levels of switchgrass ubiquitin (UBQ) transcripts (Xu *et al*., 2011). The primers used for qRT‐PCR were the same as described above for RT‐PCR (Table S1). Transcript abundance was quantified using three independent biological replicates.

### Microscopy and cell size measurement

Images of cross and longitudinal sections of fully elongated internodes were captured under Olympus IX70 Inverted Microscope with ORCA‐ER Digital Camera fluorescence optics at 10×. Images were analysed by MetaMorph Microscopy Automation and Image Analysis software to identify cell size, length and number.

### Lignin staining

For lignin staining, internode samples were collected at reproduction developmental stage (R1). Internode were cut by Vibratome series 3000 to generate 60 μm cross sections and cleared by ethanol overnight. Cleared sections were immersed in 1% chloroglucinol staining solution (in 2 : 1 ethanol/HCl) for 2 min (Baum, 2008; Bart *et al*., 2010; Wuddineh *et al*., 2015). The cross sections were placed on microscopy slides and covered by coverslip. The edges of the slides were sealed with commercial sealant and examined under Leica DM 5500B Compound Microscope with Leica DFC290 Color Digital Camera at 10×.

### GA quantification

Gibberellins were extracted in cold methanol:isopropanol:acetic acid (20:79:1, v/v/v) from samples spiked with deuterium‐labelled internal standards of GA_1_ (D2‐GA1; Olkemim Ltd, Olomouc, Czech Republic). After centrifugation at 16 000 g, the supernatants were collected and pellet extraction repeated. The pooled supernatants were evaporated and the resulting pellet re‐dissolved in 200 μL of 30% methanol. Chromatographic separation of metabolites was accomplished using a 3C18‐EP‐120 column (0.5 mm × 100 mm; Eksigent, Dublin, CA, USA) using a mobile gradient of 85% solvent A (0.1% acetic acid in HPLC‐grade water, v/v) to 95% solvent B (0.1% acetic acid in 90% acetonitrile, v/v) in 6 min at a flow rate of 15 μL/min. A 6500‐QTRAP (AB Sciex, Foster city, CA, USA) was used to acquire MS spectra. Parameters for analysis were set as follows: ESI in the negative mode (TurboIonSpray), capillary voltage −4500, nebulizer gas 25 arbitrary units (a.u.), heater gas 25 a.u., curtain gas 10 a.u., collision activation dissociation −2 and temperature 250°C. Gibberellins GA_1_ and GA_4_ were detected using multiple reaction monitoring (MRM) transitions that were optimized using the standards (GA_1_ and GA_4_; Olkemim Ltd) and the deuterium‐labelled standard. Concentrations were determined from standard curves of known GA concentrations.

### Data analysis

Comparisons between transgenic and wild‐type control plants were made by Turkey's least significant difference procedure using one‐way ANOVA and *t*‐test in SPSS software (ver.20; Chicago, IL). Standard errors are provided for statistical diagrams as appropriate. The asterisks on the bars in the figures and the tables indicate a significant difference from the wild‐type controls at *P* < 0.05 or 0.01 levels.

## Supporting information


**Figure S1** Switchgrass leaf painting using hygromycin B.

**Figure S2** Effects of *ZmGA20ox* overexpression on plant growth rate.

**Figure S3** Lignin staining of internode cross sections. (a) Wild‐type; (b) G1‐3; (c) G2‐2; (d) G3‐1; (e) G4‐1.

**Figure S4** Flowering time of *ZmGA20ox* overexpression plants.

**Table S1** Primers used in this study.
