# Supplementary figures and images for: Expression of ZmGA20ox cDNA alters plant morphology and increases biomass production of switchgrass (Panicum virgatum L.)
Source: Plant Biotechnol J. 2016 Jan 23;14(7):1532–40. doi: 10.1111/pbi.12514 (PMC5066678; doi:10.1111/pbi.12514)

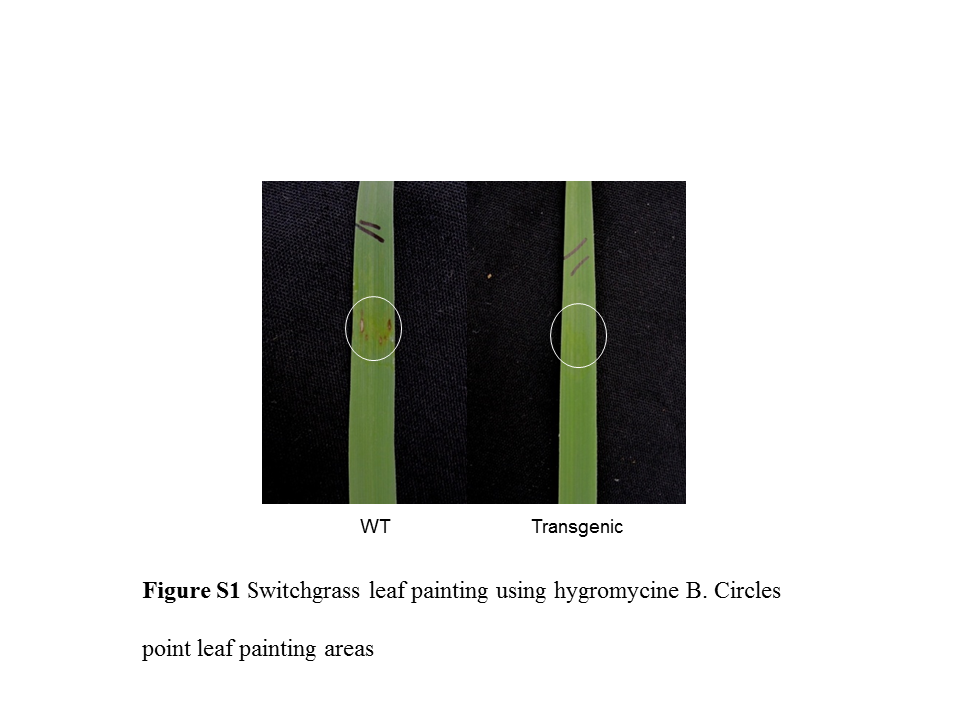

Supplement: Supplementary file 1 — Figure S1 Switchgrass leaf painting using hygromycin B. [file PBI-14-1532-s004.tif]

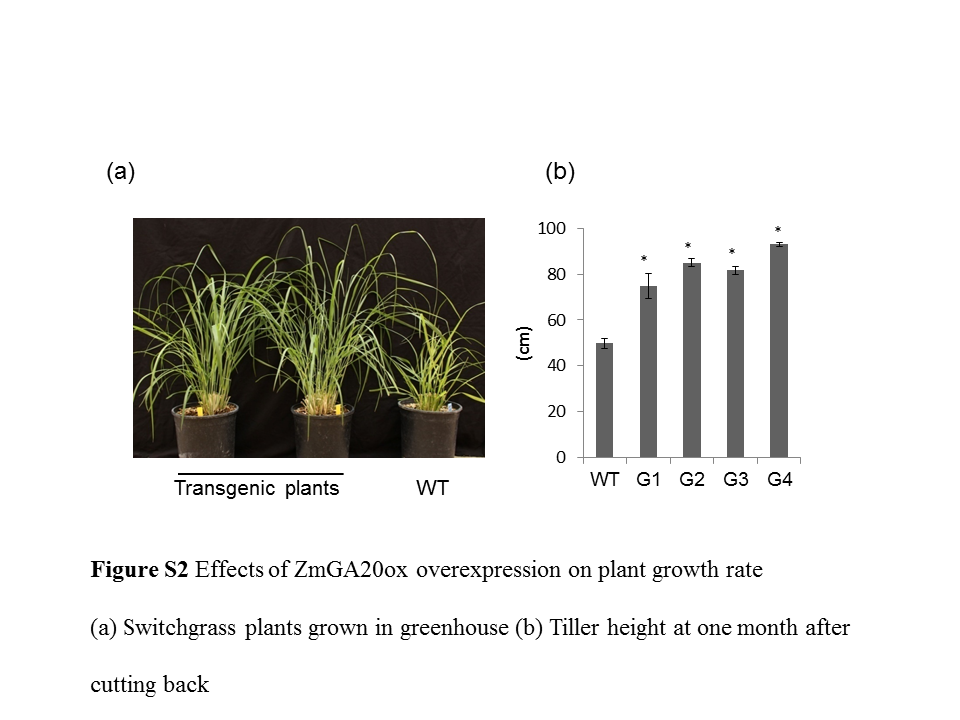

Supplement: Supplementary file 2 — Figure S2 Effects of ZmGA20ox overexpression on plant growth rate. [file PBI-14-1532-s005.tif]

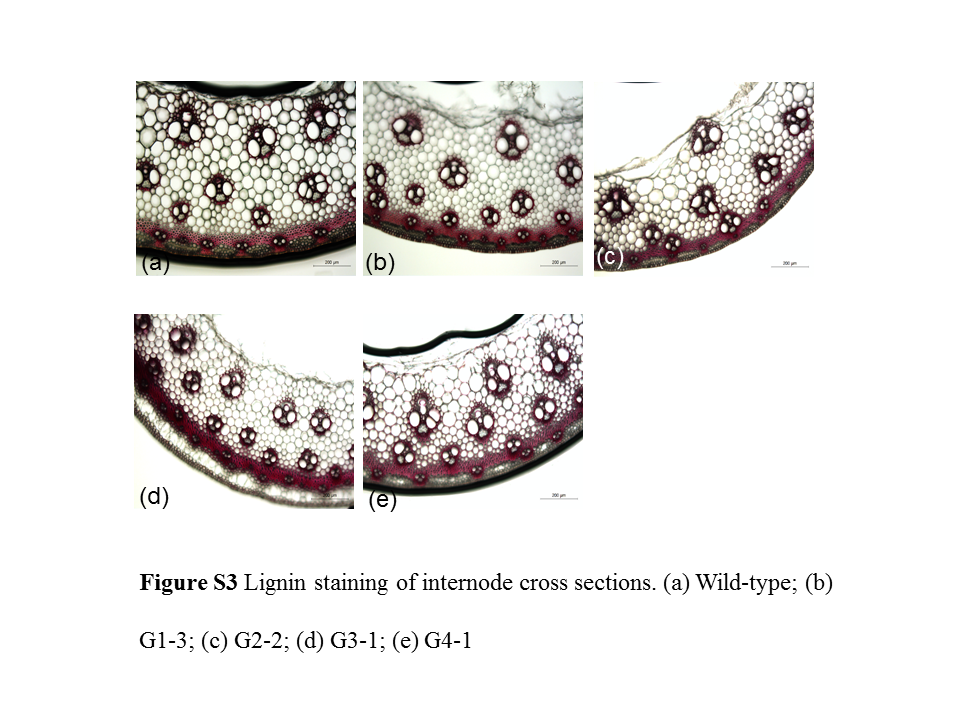

Supplement: Supplementary file 3 — Figure S3 Lignin staining of internode cross sections. (a) Wild‐type; (b) G1‐3; (c) G2‐2; (d) G3‐1; (e) G4‐1. [file PBI-14-1532-s003.tif]

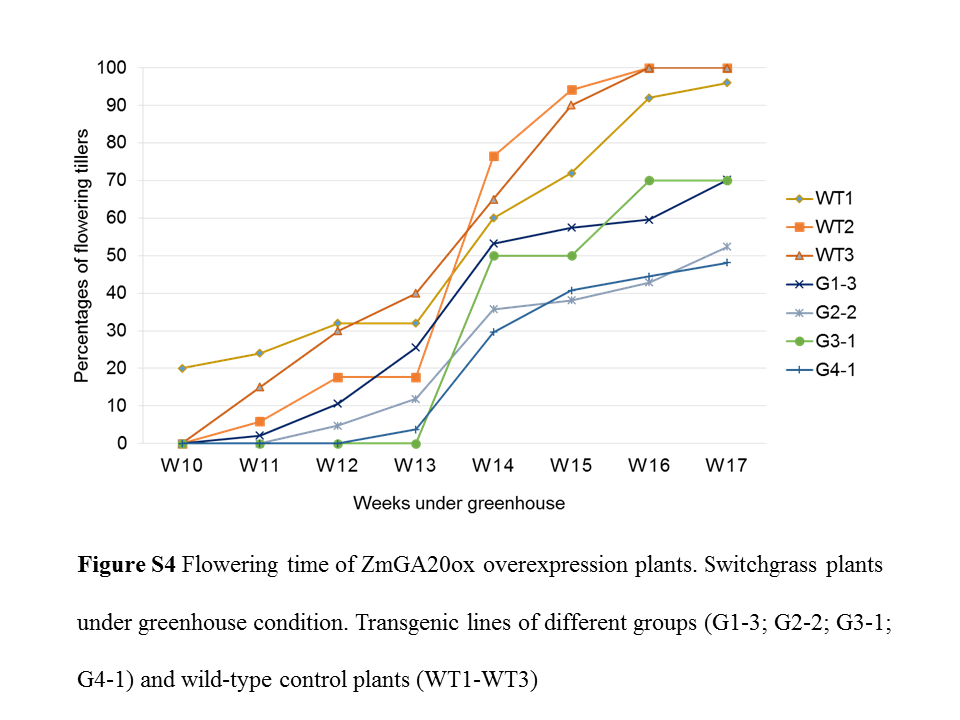

Supplement: Supplementary file 4 — Figure S4 Flowering time of ZmGA20ox overexpression plants. [file PBI-14-1532-s002.tif]
