# Supplementary material for: Expression of ZmGA20ox cDNA alters plant morphology and increases biomass production of switchgrass (Panicum virgatum L.)
Source: Plant Biotechnol J. 2016 Jan 23;14(7):1532–40. doi: 10.1111/pbi.12514 (PMC5066678; doi:10.1111/pbi.12514)
Supplement: Supplementary file 5 — Table S1 Primers used in this study. [file PBI-14-1532-s001.docx]

**Table S1** Primers used in this study

| **Primer name** | **Sequences** | **References** |
| --- | --- | --- |
|  | Primers for PCR |  |
| hptII-F | CAGGACATTGTTGGAG | Li and Qu. 2010 |
| hptII-R | TCTGTCGAGAAGTTTC | Li and Qu. 2010 |
| ZmGA20-F | GCTCTGAGATGAGCCGTCTG |  |
| ZmGA20(T)-R | ATTTGGAGAGGACACGCTCG |  |
|  | Primers for qRT-PCR |  |
| GA20-F | GAGATGGACAAGGTGGTCAG |  |
| GA20-R | GTAGTGCCTCATGGTGAAGT |  |
| Pv4CL1-F | CGAGCAGATCATGAAAGGTTACC | Shen *et al*., 2012 |
| Pv4CL1-R | CAGCCAGCCGTCCTTGTC | Shen *et al*., 2012 |
| PvCAD-F | TCACATCAAGCATCCACCATCT | Shen *et al*., 2012 |
| PvCAD-R | GTTCTCGTGTCCGAGGTGTGT | Shen *et al*., 2012 |
| PvCOMT-F | CAACCGCGTGTTCAACGA | Shen *et al*., 2012 |
| PvCOMT-R | CGGTGTAGAACTCGAGCAGCTT | Shen *et al*., 2012 |
| PvUbi-F | CAGCGAGGGCTCAATAATTCCA | Xu *et al*., 2011 |
| pvUbi-R | TCTGGCGGACTACAATATCCA | Xu *et al*., 2011 |
